# Microwave Accelerated Green Synthesis of Stable Silver Nanoparticles with *Eucalyptus globulus* Leaf Extract and Their Antibacterial and Antibiofilm Activity on Clinical Isolates

**DOI:** 10.1371/journal.pone.0131178

**Published:** 2015-07-01

**Authors:** Khursheed Ali, Bilal Ahmed, Sourabh Dwivedi, Quaiser Saquib, Abdulaziz A. Al-Khedhairy, Javed Musarrat

**Affiliations:** 1 Department of Agricultural Microbiology, Faculty of Agricultural Sciences, Aligarh Muslim University, Aligarh, India; 2 Chair for DNA Research, Department of Zoology, College of Science, King Saud University, Riyadh, Saudi Arabia; RMIT University, AUSTRALIA

## Abstract

A simple and rapid microwave assisted method of green synthesis of silver nanoparticles (AgNPs) was developed using aqueous leaf extract of *Eucalyptus globulus*(ELE), and their antibacterial and antibiofilm potential investigated. With this aim, the aqueous solutions of ELE and AgNO_3_(1 mM) were mixed (1:4 v/v), and microwave irradiated at 2450 Mhz, for 30 sec. The instant color change of the ELE-AgNO_3_ mixture from pale yellow to dark brown indicated ELE-AgNPs synthesis. The intensity of peak at 428 nm in UV-Vis spectra, due to the surface plasmon resonance of AgNPs, varied with the amount of ELE, AgNO_3_ concentration, pH and time of incubation. The biosynthesized ELE-AgNPs were characterized by UV-visible spectroscopy, XRD, TEM, SEM-EDX, FTIR and TGA analyses. The size of ELE-AgNPs was determined to be in range of 1.9–4.3 nm and 5-25 nm, with and without microwave treatment, respectively. SEM exhibited the capping of AgNPs with the ELE constituents, and validated by FTIR analysis. The FTIR data revealed the presence of plant organic constituents and metabolites bound to ELE-AgNPs, which contributes for their stability. The antimicrobial activity of ELE-AgNPs was assessed by growth and biofilm inhibition of extended spectrum β-lactamase (ESBL) producing *Pseudomonas aeruginosa*, *Escherichia coli* and methicillin-resistant *Staphylococcus aureus* (MRSA) and methicillin-sensitive *Staphylococcus aureus* (MSSA) clinical bacterial isolates. The results demonstrated that *S*. *aureus* were more sensitive to ELE-AgNPs than *E*. *coli* and *P*. *aeruginosa*. MRSA exhibited higher sensitive than MSSA, whereas *P*. *aeruginosa* were more sensitive than *E*. *coli* to ELE-AgNPs treatment. Also, significant (83 ± 3% and 84 ± 5%) biofilm inhibition was observed in case of *S*. *aureus* and *P*. *aeruginosa*, respectively. The results elucidated environmentally friendly, economical and quick method for production of colloidal bio-functionalized ELE-AgNPs, for effectual clinical applications, as broad spectrum antibacterial agents and biofilm inhibitors.

## Introduction

The phytochemicals present in plant extracts have been reported to cause reduction of metal ions to nanoparticles (NPs) and, eventually obliterate the use of toxic chemicals, high pressure, temperature, energy and maintenance of microbial cultures [1–2]. Thus, green synthesis of NPs using phytocompounds as bio-reductants is attaining a greater impetus. A variety of plant materials, such as leaf extracts, fruit, bark, fruit peels, root and callus [3] have been explored for the synthesis of NPs in different sizes and shapes. Synthesis of metal NPs is a developing research area owing to distinctive catalytic, optical, magnetic and electrical properties of NPs and their potential applications [4]. Indeed, the methods for synthesis of silver nanoparticles (AgNPs) has been increased pursued due to their antimicrobial prophylaxis in wound treatment, sterilization of medical devices, oral health protection, and food sanitation [5]. Albeit, the antimicrobial ability of AgNPs has been adequately proven, their poor stability is a primary limitation in clinical applications [6]. Stability is regarded as a critical factor when AgNPs are to be used as antibacterial agents, however, limited studies have been perform on improving their stability and antibacterial properties. Panacek et al. [7] have demonstrated that the stability and protracted antibacterial action of AgNPs depends on their tendency to aggregate and release of silver ions. Surface modification of AgNPs with citrate, polyvinylpyrrolidone, and amino acids has been reported to obtain functional stability [8–10]. Furthermore, Qu et al. [11] suggested that AgNPs coated with *Agrimoniae herba* extract as reducing agent and stabilizer, confer better stability and pharmacological activity as compare to bare AgNPs.

Various techniques for AgNPs synthesis such as surface adsorption and deposition, arc discharge, laser chemical vapor deposition, plasma and emulsion polymerization have been reported [12]. Most of these methods employ toxic chemicals as reducing and stabilizing agents, organic solvents or non-biodegradable agents [12], which may pose environmental risks due to use of hazardous chemicals [13]. Therefore, the alternatives methods, which are less hazardous and economically viable are being explored. AgNPs have also been synthesized by using microorganisms and plant extracts including *Hibiscus rosa sinensis*, *Azadirachta indica*, black tea, *Emblica officinalis*, *Cinnamomum camphora*, *Cinnamomum zeylanicum*, *Parthenium Camellia sinensis*, *Magnolia kobus*, *Diopyros kaki*, *Geranium*, natural rubber, *Alfalfa*, aloe [14] and *Morganella psychrotolerans* leave extracts [15]. Recently, silver and iron NPs of various sizes and morphologies have been synthesized by using coffee and green tea extract [14]. Dubey et al. [16] and Sulaiman et al. [17] have reported the extracellular biosynthesis of AgNPs with *Eucalyptus hybrida* and *E*. *chapmaniana* leaf methanolic extracts. In spite of extensive studies in this area, the potential of the plants as biological materials for the synthesis of NPs is currently under exploitation. Indeed, the synthesis of NPs using plant extract is potentially advantageous over microorganisms due to the ease of biohazards and the culture of the microorganisms.

In the present study, we report the synthesis of AgNPs, using aqueous leaf extract of *Eucalyptus globulus*, an aromatic tree, belonging to a large genus of the Myrtaceae family, [18]. It has been extensively cultivated in many countries, including India [19], and can grow on a wide range of substrates in moderately fertile loams and well-drained soil. Specifically, silver has been chosen because silver in NP or ionic form is most toxic for microorganisms, as compared to other metals [20]. Also, the nanosized silver has been shown to exert efficient toxicity to microbes with a lesser toxicity in mammalian cells [21] and its antimicrobial properties are well-established [22]. In this paper, we presents a rapid and simple synthesis of *Eucalyptus globulus* extract (ELE)-capped AgNPs by reduction of AgNO_3_ with ELE and without involving any supplementary chemicals or physical steps like centrifugation, sonication, annealing, etc. We have assessed the effects of (i) leaf extract quantity, (ii) concentration of metal solution, (iii) pH, and (iv) contact time, to optimize the method of synthesis. The stability of ELE-AgNPs was evaluated and antibiofilm and antibacterial activities determined based on growth inhibition, minimum inhibitory concentration (MIC) and MBC concentrations, against the extended spectrum β-lactamase (ESBL) producing *Pseudomonas aeruginosa*, *Escherichia coli* and Gram-positive methicillin-resistant *Staphylococcus aureus* (MRSA) and methicillin-sensitive *Staphylococcus aureus* (MSSA) clinical bacterial isolates.

## Materials and Methods

*Eucalyptus globulus* leaves were collected from the tree planted in the university campus of Aligarh Muslim University (AMU), Aligarh (latitude and longitude coordinates: 27.921763, and 78.075866). Silver nitrate (AgNO_3_; 99.8%) was purchased from Glaxo laboratories (India) Ltd, Mumbai. Luria Bertani broth (LB), nutrient broth (NB), Muller-Hinton (MH) broth and brain heart infusion (BHI) broth were obtained from Hi Media, Mumbai, India. All glass wares were purchased from Borosil, India. The glass wares were treated with 1 N H_2_SO_4_, and washed thoroughly with tap water and then with deionized ultrapure water (18.2MΩxcm) (TKA Genpure, Niederelbert, Germany). The bacterial isolates including *E*. *coli-157*, *Pseudomonas aeruginosa-620* (Extended-Spectrum β-Lactamases)**,** Methicillin-resistant *Staphylococcus aureus* MR-6 (MRSA) and Methicillin-sensitive *Staphylococcus aureus* (MSSA) MS-6, and ESBL negative *E*. *coli-255* and *Pseudomonas aeruginosa-222* were obtained from the culture stocks of the Department of Microbiology, Jawaharlal Nehru Medical College, AMU, Aligarh, India, and revalidated in our laboratory. The strains were sub-cultured in LB, NB, MH and BHI broths and maintained on agar plates of their respective medium. The cultures were stored at -20°C in 20% glycerol, for long term preservation.

### Preparation of aqueous *E*. *globulus* leaf extract [ELE]

The fresh and healthy leaves of *E*. *globulus* were thoroughly washed with tap water and then with deionized water. The leaves were air dried and chopped into small pieces. Chopped leaves (20g) were added in 100 ml deionized autoclaved water in a beaker, and heated at 90°C on a temperature controlled water bath for 10 min. The extract was cooled and filtered through Whatman paper No.1 filter paper, and stored at 4°C until used.

### Synthesis of ELE-capped AgNPs

The reaction conditions for synthesis of ELE-AgNPs were standardized, and finally 3 ml of ELE was mixed with 12 ml of 1mM AgNO_3_, pH 8.0 in a 100 ml conical flask. For rapid microwave assisted synthesis, the ELE-AgNO_3_ reaction mixture was subjected to microwave treatment in a domestic microwave oven (IFB 20PG3S) operating at a power of 800W and frequency 2450 MHz, for a short pulse of 30 sec. The mixture was then allowed to stand at room temperature. The reaction conditions were optimized by performing experiments with different sets of flasks, wherein the reactions of ELE- AgNO_3_ were carried out as a function of the amount of ELE (1–5 ml), AgNO_3_ (0.1–1 mM), pH (4–10), and time of incubation (0–30 min) for microwave assisted synthesis. ELE-AgNPs synthesis was also performed at elevated temperature by incubating the reaction mixture in water bath at 60°C for 30 min. However, the conventional ELE-AgNPs synthesis was performed by incubation of reaction mixture for 3 h without microwave treatment. All reactions were carried out in dark at ambient temperature. The flask containing same amount of ELE without AgNO_3_ was run as a parallel control under identical conditions. A drastic change in color from pale yellow to dark brown was observed. The colored solution was then dried in a vacuum oven (NSW, Delhi, India) at 50°C for 24 h. The dark brown powder obtained was stored in acid cleaned glass vials for further characterization by UV-Vis, XRD, TEM, SEM-EDX, and FTIR analyses.

### Characterization of ELE-AgNPs

#### UV-visible spectroscopy

The biosynthesized colloidal ELE-AgNPs were analyzed for surface plasmon resonance by use of a double beam UV-Vis spectrophotometer (UV5704S from Electronics, India ltd) in the wave length range of 350–600 nm, at a resolution of 0.5 nm. The spectra of microwave assisted ELE-AgNPs obtained under different experimental conditions with varying ELE, AgNO_3_, pH, were recorded periodically at intervals of 5, 10, 15, 20, 25 and 30 min. However, the spectra of conventionally synthesized ELE-AgNPs were recorded at 1, 2 and 3 h. Deionized water was used as a blank and the background absorption was subtracted from UV-Vis spectrum of ELE. To determine the stability of ELE-AgNPs, the spectral measurements were extended up to 4 weeks at regular intervals of 1 week.

#### X-ray diffraction measurements

The X-ray diffraction (XRD) pattern of powdered sample of ELE-AgNPs was recorded on MiniFlex II benchtop XRD system (Rigaku Corporation, Tokyo, Japan) operating at 40 kV and a current of 30 mA with Cu Ka radiation (k = 1.54 Å). The diffracted intensities were recorded from 20° to 80° 2Ɵ angles. The crystalline size of the AgNPs was calculated following the Debye–Scherrer’s formula: D = 0.9λ/βcosθ: whereas, D is the crystal size of AgNPs, λ is the wavelength of X-ray source used (1.541^O^A), *β* is the full-width-at-half-maximum of the diffraction peak [23].

#### Scanning electron microscopy [SEM, SEM-EDX] and ELE-AgNPs-bacterial interactions

Scanning electron microscopic analysis was carried out using fine powder of the ELE-AgNPs on a carbon tape in JSM 6510LV scanning electron microscope (JEOL, Tokyo, Japan) at an accelerating voltage of 15 kV. The elemental analysis of ELE-AgNPs was performed using Oxford Instruments INCAx-sight EDAX spectrometer equipped SEM. Also, the interaction studies for observing the morphological changes in *E*. *coli* and *S*. *aureus* cells up on treatment with ELE-AgNPs and untreated control cells were performed by use of SEM following the methods of Ansari et al. [24]. Briefly, the samples containing untreated and treated bacterial cells were deposited on a Millipore filter (Millipore). Bacterial cells (10^6^ CFU/ml) were treated with 20 μg/ml ELE-AgNPs for 6 h at 37°C and centrifuged at 3000 rpm for 10 min. The pellets were washed with phosphate-buffered saline [PBS] three times and pre-fixed with 2.5% glutaraldehyde for 1 h at 4°C. The pre-fixed cells were washed with PBS two times and post-fixed with 1% osmium tetroxide for 1 h at 25°C. After three successive washes with PBS, the samples were dehydrated by sequential treatment with 30, 50, 70, 80, 90 and 100% of ethanol for 10 min each. The cell biomass was then fixed and the coated samples were observed under scanning electron microscope (SEM) (JEOL, Tokyo, Japan) at an accelerating voltage of 10–15 kV.

#### Transmission Electron Microscopy [TEM]

Transmission electron microscopy was performed on JEOL 100/120 kV TEM (JEOL, Tokyo, Japan) with an accelerating voltage of 200 kV [25] transmission electron microscope. Samples for microscopy were prepared by dropping 10μl of ELE-AgNPs sample on a copper grid. Excess solution was removed by using a piece of soft filter paper. The copper grid was then dried for 6 h in an oven at 80°C.

#### Fourier Transform Infrared [FTIR] spectroscopy

FTIR was employed for assessment of functional groups on ELE and ELE-AgNPs, separately. Briefly, the air dried powder of ELE and ELE-AgNPs were diluted with spectroscopic grade KBr (mass ratio of about 1:100) and the spectra were recorded. FTIR measurements were carried out on Perkin Elmer FT-IR spectrometer Spectrum Two (Perkin Elmer Life and Analytical Sciences, CT, USA) in the diffuse reflectance mode at a resolution of 4 cm ^−1^ in KBr pellets [26].

#### Thermogravimetric analysis

The thermogravimetric analysis (TGA) for functionalization and thermal stability of the ELE-AgNPs was performed by use of a Perkin Elmer Pyris 1 TGA Thermogravimetric Analyzer at a heating rate of 10°C/min under nitrogen atmosphere, following the method of Rao et al. [27].

### Antibacterial effect of ELE-AgNPs by well diffusion method

ELE-AgNPs were analyzed for their antibacterial activity. In brief, 0.1 ml of exponentially grown cultures (1–2×10^8^ cells/ml) *E*. *coli*, *P*. *aeruginosa* (Extended-spectrum β-Lactamases), Methicillin-resistant *Staphylococcus aureus* (MRSA) and Methicillin-sensitive *Staphylococcus aureus* (MSSA) were uniformly spread on LA plates. Well of 4 mm diameter were cut on plates and sealed with soft agar (0.7%) prior to adding test solution. Subsequently, increasing amounts (25, 50, and 100μl) of ELE-AgNPs were transferred to the wells on the plates with the help of a micropipette. The plates were incubated at 37°C for 24 h and sizes of growth inhibition zones were determined by measuring the radius of the zones.

#### Determination of minimum inhibitory and minimum bactericidal concentrations of ELE-AgNPs

Minimum inhibitory concentration (MIC) and minimum bactericidal concentration (MBC) values of ELE-AgNPs against *E*. *coli*, *P*. *aeruginosa* (ESBL), MRSA, and MSSA isolates were determined following the serial two-fold dilutions procedure, described by Ansari et al. [24]. Briefly, the bacterial inoculums of ~2 x 10^7^ CFU/ml were treated with ELE-AgNPs in concentrations ranging from 3 to 30 μg/ml. The treated and untreated control samples were incubated for 24 h at 37°C. The MIC was defined as the lowest concentration of antimicrobial agents that yielded no visible growth of the microorganisms [24]. For MBC determination, 100 μl aliquots from the culture tubes in which no visible bacterial growth was observed were spread on the LA plates without ELE-AgNPs. The plates were then incubated for 24 h at 37°C. The MBC endpoint is defined as the lowest concentration of antimicrobial agent that kills 100% of the initial bacterial population [24].

#### Bacterial growth inhibition assay

The effect of ELE-AgNPs and ELE alone on the growth of *E*. *coli*, *P*. *aeruginosa* (ESBL), MRSA, and MSSA isolates was assessed by the optical density (OD 620 nm) measurements of treated and untreated bacteria. The aliquots of 100 μl each from freshly grown cultures of *E*. *coli*, *P*. *aeruginosa* (ESBL) and *S*. *aureus* (MRSA and MSSA) were inoculated in 5 ml LB in glass test tubes maintaining aseptic conditions. To this ELE-AgNPs were added to achieve the increasing concentrations of 3 to 30 μg/ml. Untreated cells were used as control. The treated and untreated samples (100 μl each) were transferred to a microtitre plate and incubated at 37°C. The absorbance was measured at 620 nm at regular intervals of 2 h by use of a microplate reader (Thermo scientific Multiskan EX, REF 51118170, China).

### Effect of ELE-AgNPs on biofilm formation

Microtitre plate assay was performed following the method of Dwivedi et al. [28] to determine the effect ELE-AgNPs on biofilm formation by *P*. *aeruginosa* and *S*. *aureus*. Briefly, the wells of sterile polystyrene microtitre plate were seeded with 100 μl of freshly grown bacterial cells (1 x 10^7^ CFU/ml) in LB. To each well ELE-AgNPs were added in increasing concentrations in the range of 6–30 μg/ml. The plates were incubated at 37°C for 24 h. The bacterial suspension was discarded and the wells were washed with autoclaved phosphate buffer saline (PBS). Wells were then stained with 200 μl of 0.25% crystal violet and incubated again at 37°C for 30 min. Further, the wells were washed, air dried, and bound stain was solubilized in 200 μl of 95% ethanol. The absorbance was read at 620 nm by use of a microplate reader (Thermo scientific Multiskan EX, REF 51118170, China).

## Results and Discussion

### Microwave assisted green synthesis of ELE-AgNPs

**Fig 1:**
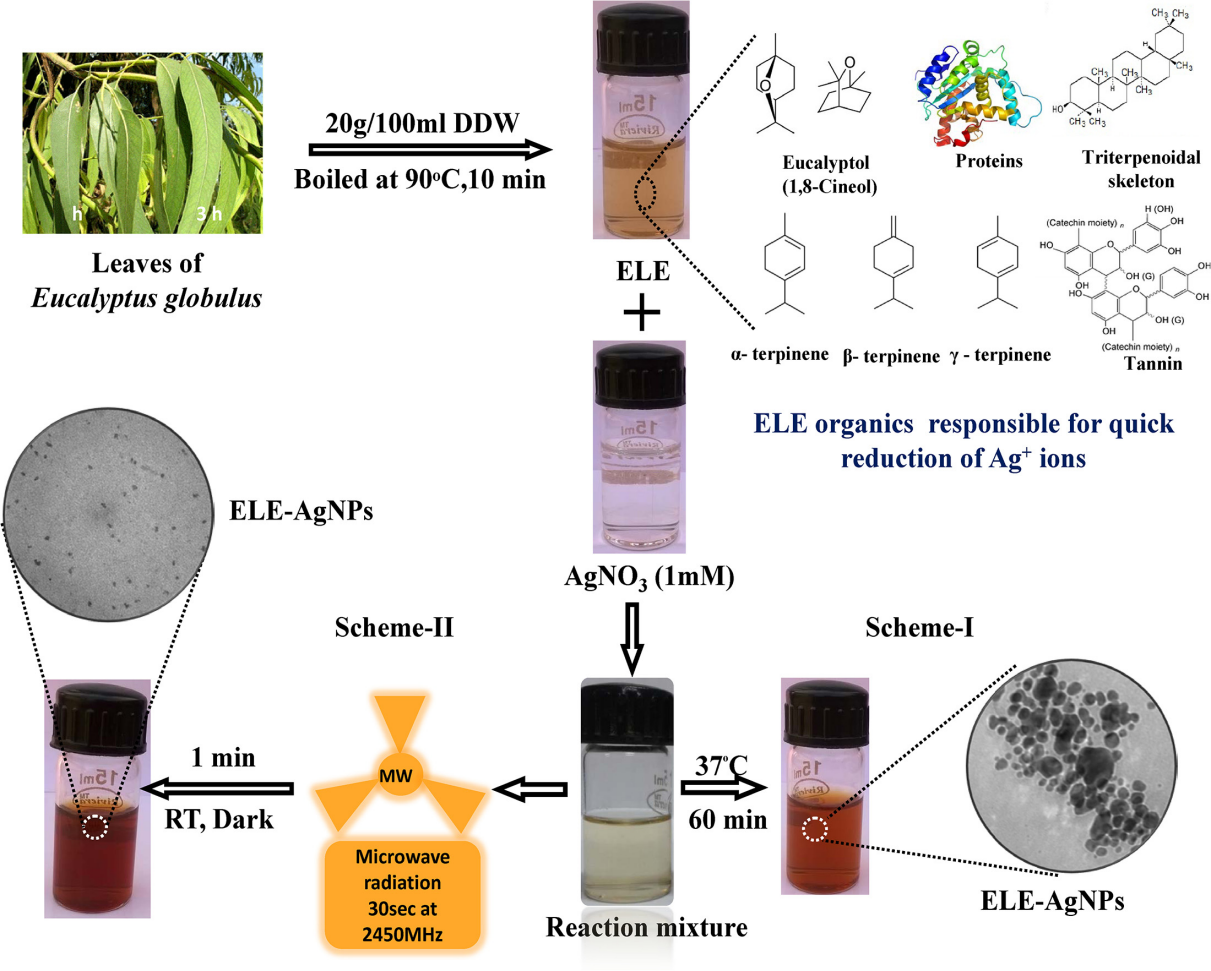
Graphical representation of ELE-AgNPs synthesis depicting scheme-I and-II.

**Fig 2:**
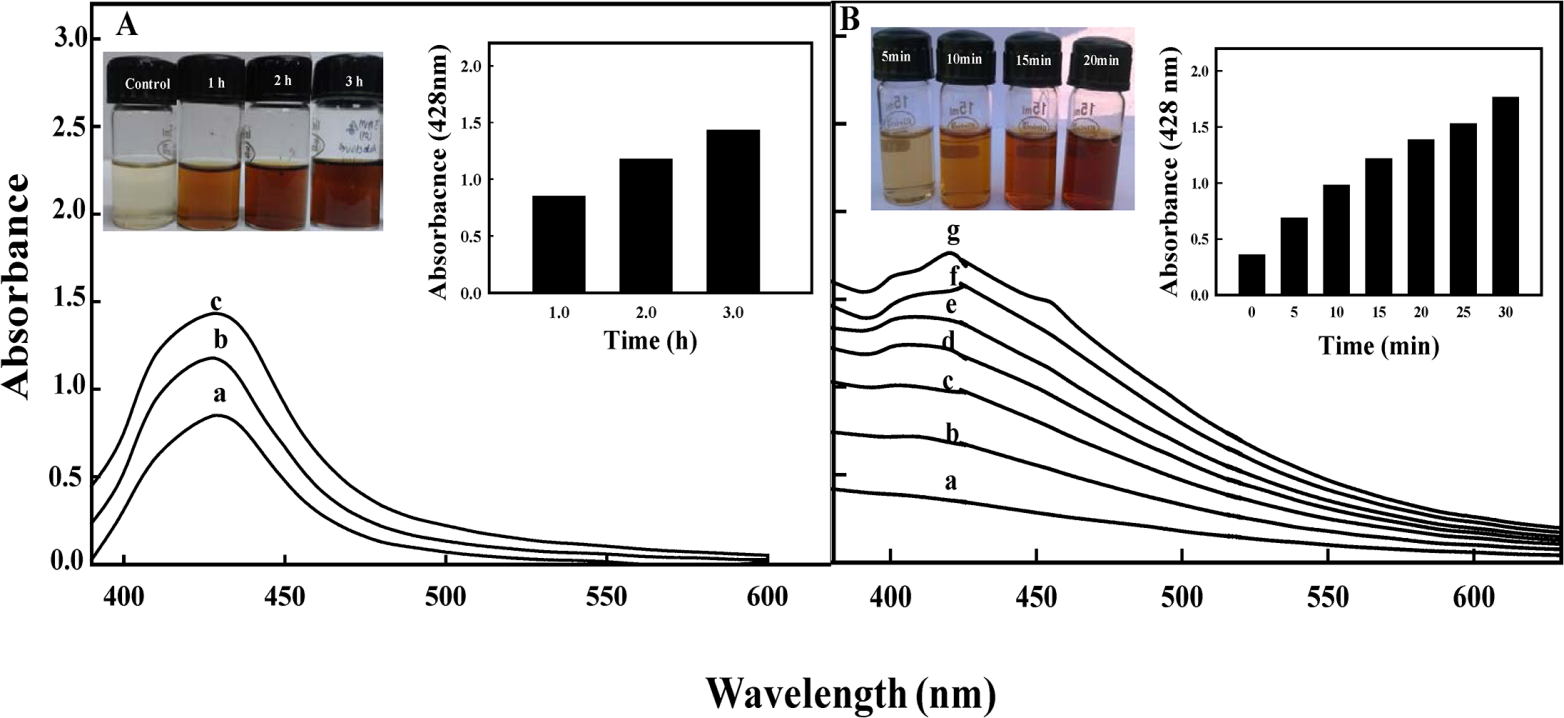
UV-vis absorption spectra of ELE-AgNPs. Panels show absorbance measurement during ELE-AgNPs synthesis as a function of time, as: (A) without microwave irradiation; a—c represents incubation time as 1, 2 and 3 h at 37°C, and (B) with microwave irradiation for 30 sec, followed by incubation in dark; a-g represents 0, 5, 10, 15, 20, 25 and 30 min at ambient temperature.

The biomimetic and green synthesis has lately developed as the environmentally safe and cleaner methods of NPs synthesis, involving a variety of biological entities such as bacteria [29], actinomycetes [30], fungi [31], cyanobacteria [32], as well as biomolecules [33] and plant tissues. However, the plant mediated synthesis of NPs is a relatively under exploited field, and is gaining wider attention. In this study, *Eucalyptus globulus* leaf extract (ELE) has been used for the synthesis of ELE functionalized AgNPs. The schematic representation of the approaches of ELE-AgNPs synthesis is depicted in the scheme in Fig 1. The scheme-I shows that the aqueous solutions of ELE and AgNO_3_ when mixed in a ratio of 1:4, exhibited a gradual change in color from pale yellow to dark brown after 3 h at 37°C. However, the scheme-II depicts that the microwave irradiation of the identical reaction mixture at 2450 Mhz for a short pulse of 30 s, followed by 30 min incubation at ambient temperature, resulted in instant change in color of the ELE-AgNO_3_ reaction mixture to dark brown, which is regarded as a preliminary indication of AgNPs synthesis. The peak at 428 nm in UV-Vis spectra (Fig 2A and 2B) is attributed to the surface plasmon resonance of AgNPs formed by reduction of aqueous Ag ions and its intensity increased as a function of time. The change in color was due to collective oscillation of the conduction electrons of the ELE-AgNPs produced in the reaction mixture, which increases steadily with time of exposure. However, no absorption peak corresponding to the controls ELE and/or Ag ion solution, in the range of measurement was observed. Also, the plasmon band has been symmetric, which indicates that the solution does not contain much of aggregated particles.

Furthermore, the results demonstrated that a composite approach of microwave assisted green synthesis significantly accelerates the process of ELE-AgNPs synthesis. Fig 2A and 2B shows the kinetics of ELE-AgNPs synthesis without and with microwave treatment in an aqueous solution with the time-dependent change in the onset absorbance at intervals of 1, 2, and 3 h and 5, 10, 15, 25 and 30 min, respectively. Furthermore, ELE-AgNPs synthesis at an elevated temperature of 60°C by conventional heating method also occurred within 10 min (S1 Fig), which was comparable with the microwave assisted synthesis. Most likely, the faster rate of reaction with microwave irradiation is due to rapid and uniform heating of the reaction medium, which resulted in homogeneous nucleation and growth of NPs. (Fig 2B). Since heating in a microwave is generated by interaction of the permanent dipole moment of the molecule with high frequency (2.45 GHz) electromagnetic radiations, it is reported to reduce the reaction time by a factor of ~ 20 as compared to conventional heating [34]. Our results support the observations of Joseph and Mathew [35], who have recently reported that integrating biosynthetic methods with microwave chemistry significantly enhance the process of NPs synthesis without affecting the green reaction condition. Also, Kudle et al. [36] demonstrated the microwave assisted AgNPs synthesis using flower extract of *Boswellia serrata* and orange peel extract, respectively. Indeed, as a zero-order approach, the typical synthesis of nanoparticles could be divided into three distinct stages: nucleation, evolution of nuclei into seeds, and growth of seeds into nanocrystals. The final shape of a nanocrystal is determined primarily by the internal structure of the corresponding seed and the binding affinity of the capping agent. Brust et al [37] have suggested a competition between the capping and the growth of a particle and that the particle size is controlled by the surface coverage, not by the reduction kinetics of precursors. Thus, larger the rate of capping, smaller is the particle size. Gurunathan et al [38, 39] have also demonstrated that at room temperature, AgNPs of 50 nm are synthesized, whereas at 60°C AgNPs of 15 nm are synthesized. Furthermore, Deepak et al [40] have suggested that at lower temperature and pH, less nucleation occurs thereby forming larger particles whereas at higher pH and temperature more nucleation may occur thus forming smaller particles. Therefore, the size of NPs under our experimental conditions with microwave treatment (scheme-II) was smaller than the NPs formed by conventional method at room temperature (scheme-I) because the extent of nucleation and capping might be faster with microwave treatment than the synthesis at room temperature. As the temperature increase, the dynamics of the ions increase and more nucleation regions are formed due to the availability of−OH ions and increased temperature. The conversion of Ag^+^ to Ag° increases followed by increase in the kinetics of the deposition of the silver atoms. Unlike the classical LaMer mechanism for particle formation [41], the nanocrystal formation can be controlled by continuous nucleation, growth, and capping of particles throughout the synthesis [42–43]. It has been reported that in a constant synthesis environment, continuous nucleation-growth-capping mechanism leads to complete capping of particles (no more growth) at the same size, while the new ones are formed continuously leading to synthesis of more monodisperse particles [43].

**Fig 3:**
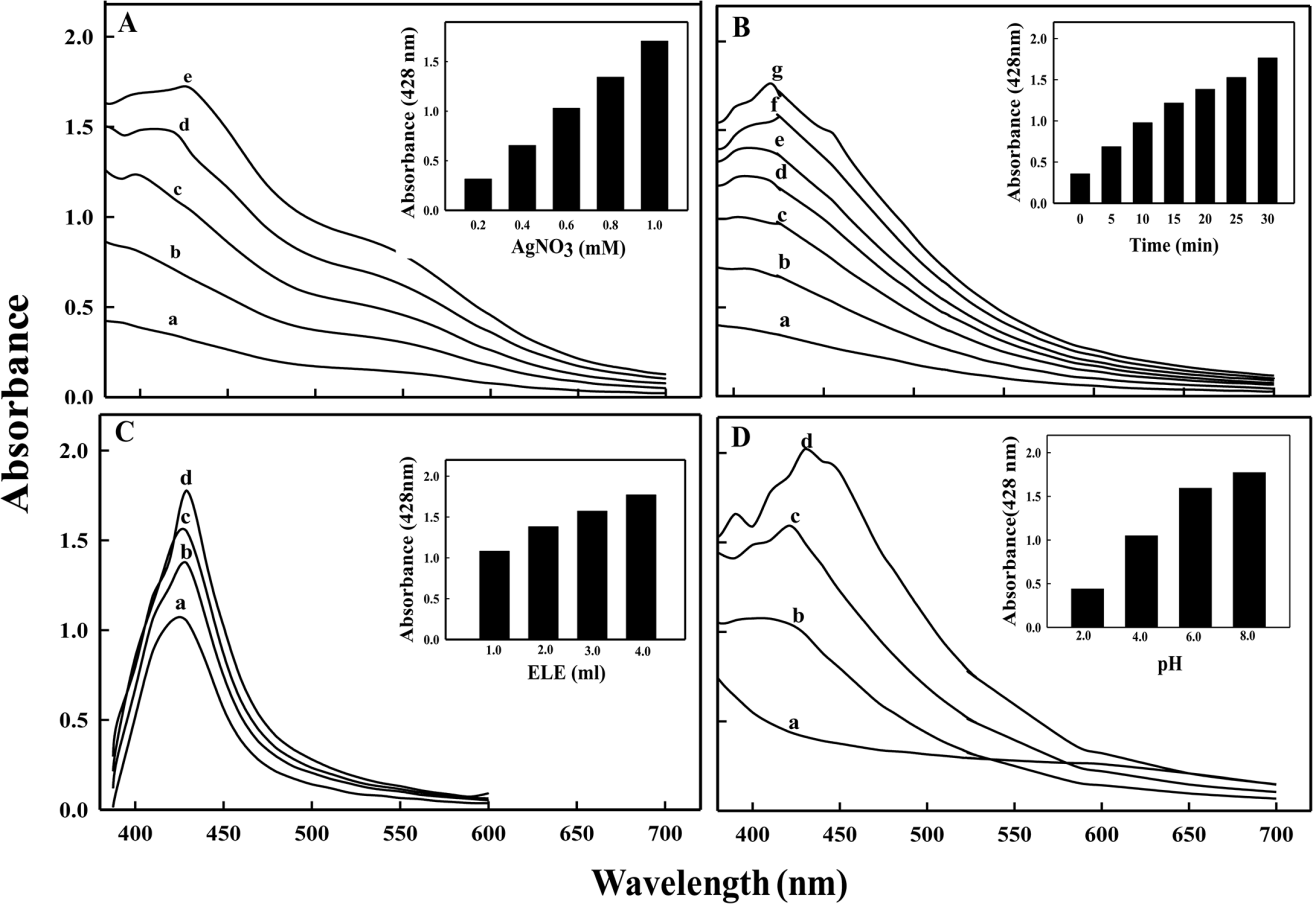
UV-vis absorption spectra of ELE-AgNPs synthesized by microwave assisted method. Spectra under different reaction conditions during synthesis were recorded as a function of: (A) AgNO_3_ concentration (a-e as 0.2, 0.4, 0.6, 0.8 and 1.0 mM) in presence of 1 ml ELE; (B) time (a-g as 0, 5, 10, 15, 20, 25 and 30 min in presence of 1:4 v/v ELE-AgNO_3_); (C) ELE (a-d as 1, 2, 3 and 4 ml) with 1 mM AgNO_3_; and (D) pH (a-d as 2.0, 4.0, 6.0 and 8.0) in 1:4 v/v ELE-AgNO_3_ reaction mixture.

The results in Fig 3A–3D exhibit the optimization of conditions for microwave assisted ELE-AgNPs synthesis. The spectra revealed that the values of absorbance steadily increased as a function of AgNO_3_ concentration (0.2–1.0 mM), reaction time (0–30 min), pH (4.0–10.0), and ELE ratio (1:1 to 1:4 v/v AgNO_3_) at ambient temperature. The results indicate increased reduction of AgNO_3_ by aqueous ELE with increasing pH of the reaction mixture. This is attributed to the availability of more H^+^ from the metabolites at higher pH, which enables quicker reduction of AgNO_3_, and oxidation of the metabolites [44]. It is, therefore, suggested that the microwave assisted synthesis using ELE extracts, as both reducing and capping agent, could be exploited for rapid and one-pot green synthesis of ELE-AgNPs, due to short reaction time, lower energy consumption, relatively smaller size and better product yield, as has also been reported by Nadagouda et al. [45].

The ELE-AgNPs were quite stable as no significant change in the absorbance of the colloidal ELE-AgNPs solution occurred up to six months (S2 Fig). The comparative spectra in S2 Fig demonstrate the prolonged stability compared to the unstabilized AgNPs. The stability of ELE-AgNPs is attributed to the presence of alkaloids, tenins, triterpenoids, flavonoids, proteins, carbohydrates and metabolites present in ELE [16], which may acts as capping agent in the reaction milieu and stabilizes the AgNPs by developing a steric hindrance around the particles, and preventing aggregation of the NPs due to electrostatic interactions. This is supported by the observation that the SPR band of ELE-AgNPs did not show any noticeable shift in its position even when the UV-vis. spectra were recorded after storage at room temperature for several months.

### Characterization of ELE-AgNPs

**Fig 4:**
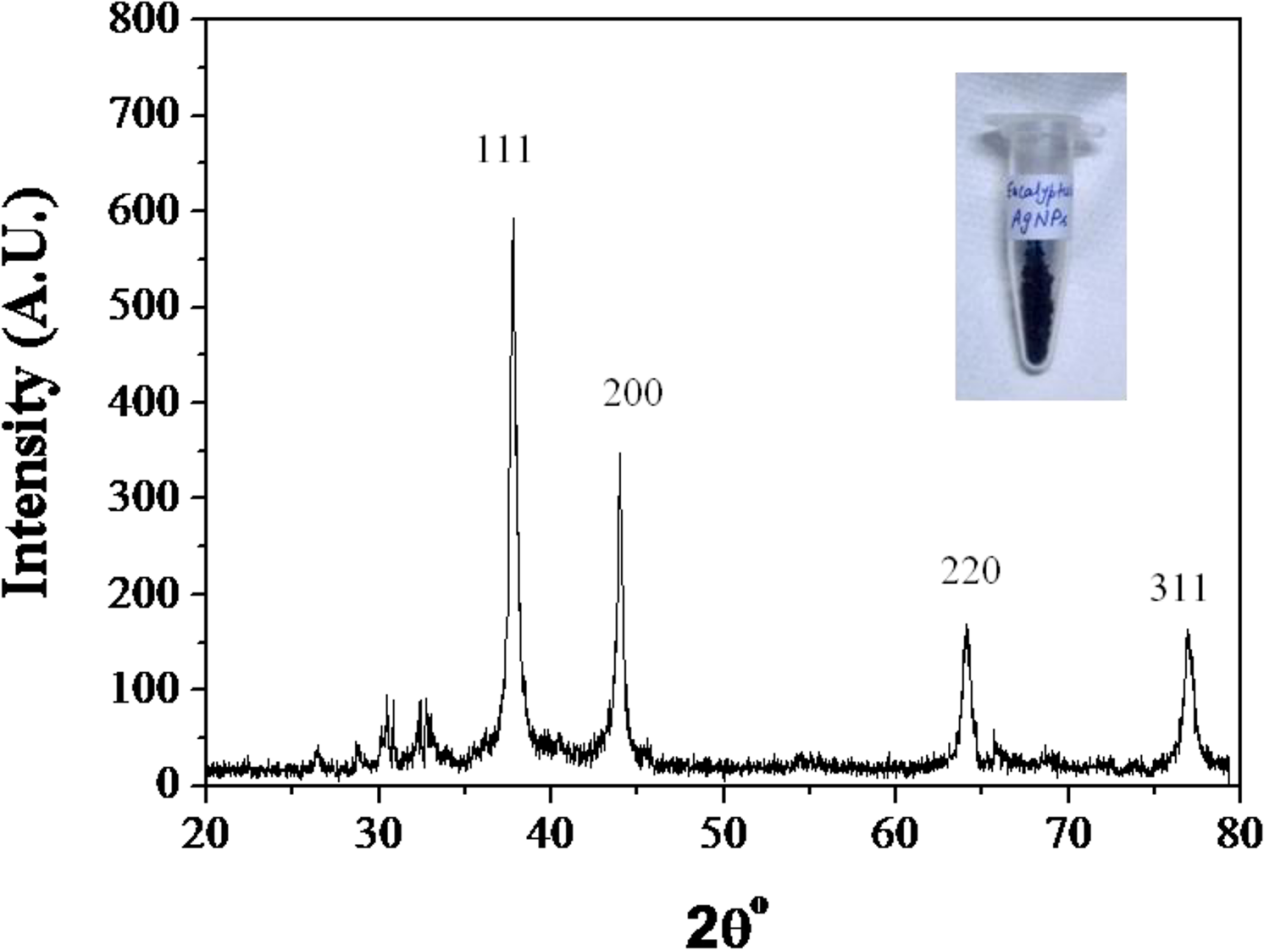
X-ray pattern of ELE-AgNPs synthesized following the scheme-I.

Fig 4 shows the XRD pattern obtained for the extracellular ELE-AgNPs with four intense peaks in the spectrum of 2θ values ranging from 20 to 80. The diffractions at 38°, 44.18°, 64.29° and 77.19° can be indexed to the [111], [200], [220] and [311] planes of the face centered cubic (fcc) silver, respectively and suggest the crystalline nature of ELE-AgNPs (JCPDS File No. 03–0921). Biosynthesized AgNPs using different fungi have also exhibited crystalline nature [46, 47]. The average particle size of the ELE-AgNPs was determined to be 18 nm, based on `full-width-at-half-maximum (FWHM) value for (111) plane of reflection. Dubey et al. [16] have also reported the extracellular biosynthesis of AgNPs (50–150 nm) at ambient temperature using methanolic extract of *Eucalyptus hybrida* leaf, which were much larger in size than the ELE-AgNPs produced by our methods using aqueous extracts of *Eucalyptus hybrida* leaf. Indeed, the biosynthesized NPs are reported to exhibit significant size variability depending on the factors like temperature, pH and type of plant extracts [46]. Synthesis of AgNPs in size ranges of 5–30, 25–40, 50–100, 16–40, 15–150 nm has been reported with leaf extracts of *Mentha Piperita*, *Azadirachta indica*, *Pelargonium graveolens*, *Eucalyptus hybrida* [14,16], respectively.

**Fig 5:**
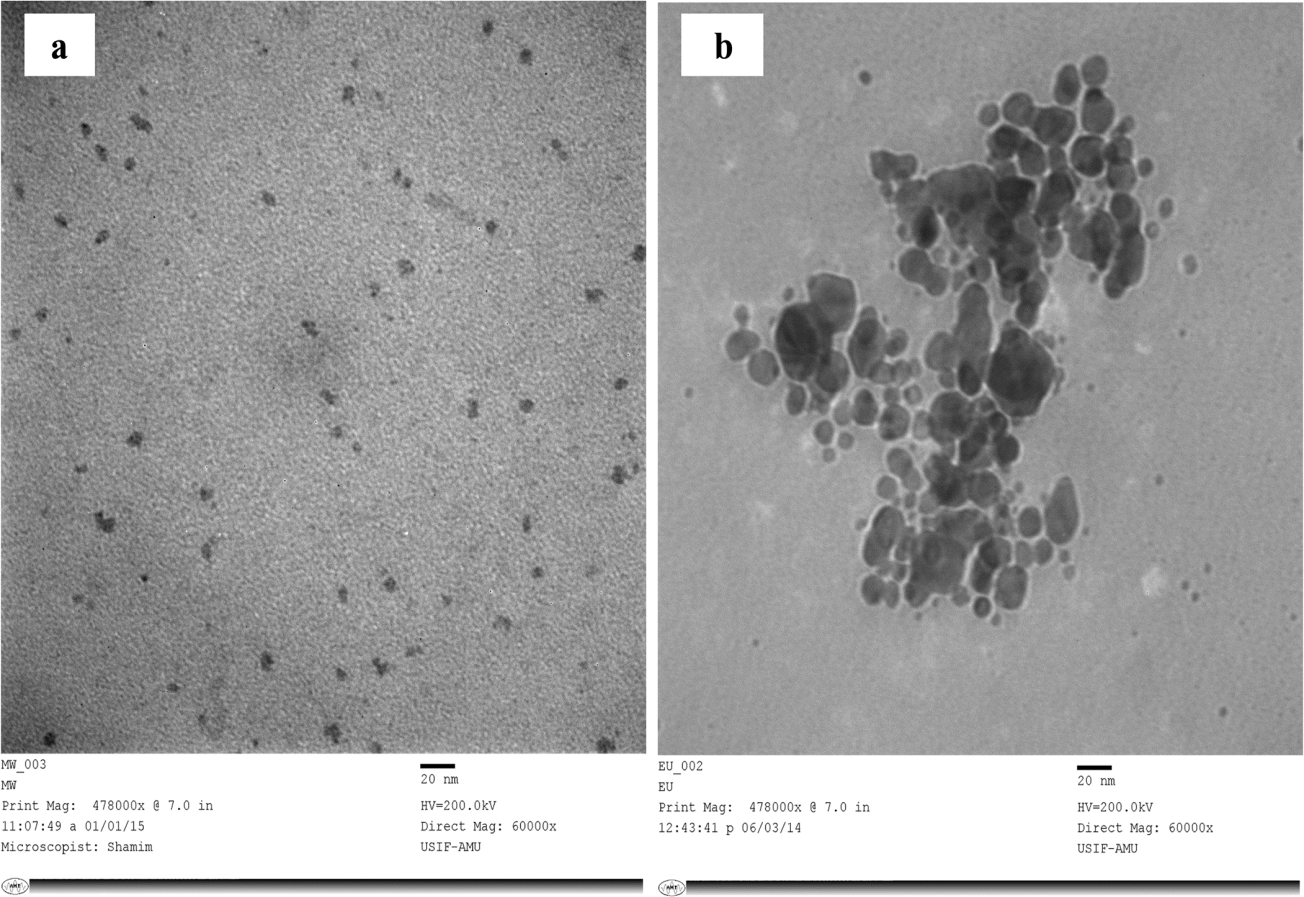
TEM micrograph of synthesized ELE-AgNPs. Panel (A) depicts the TEM images of ELE-AgNPs synthesized by microwave assisted approach, as specified in Fig 1, scheme-II; Panel (B) shows the images of ELE-AgNPs synthesized at 37°C.

**Fig 6:**
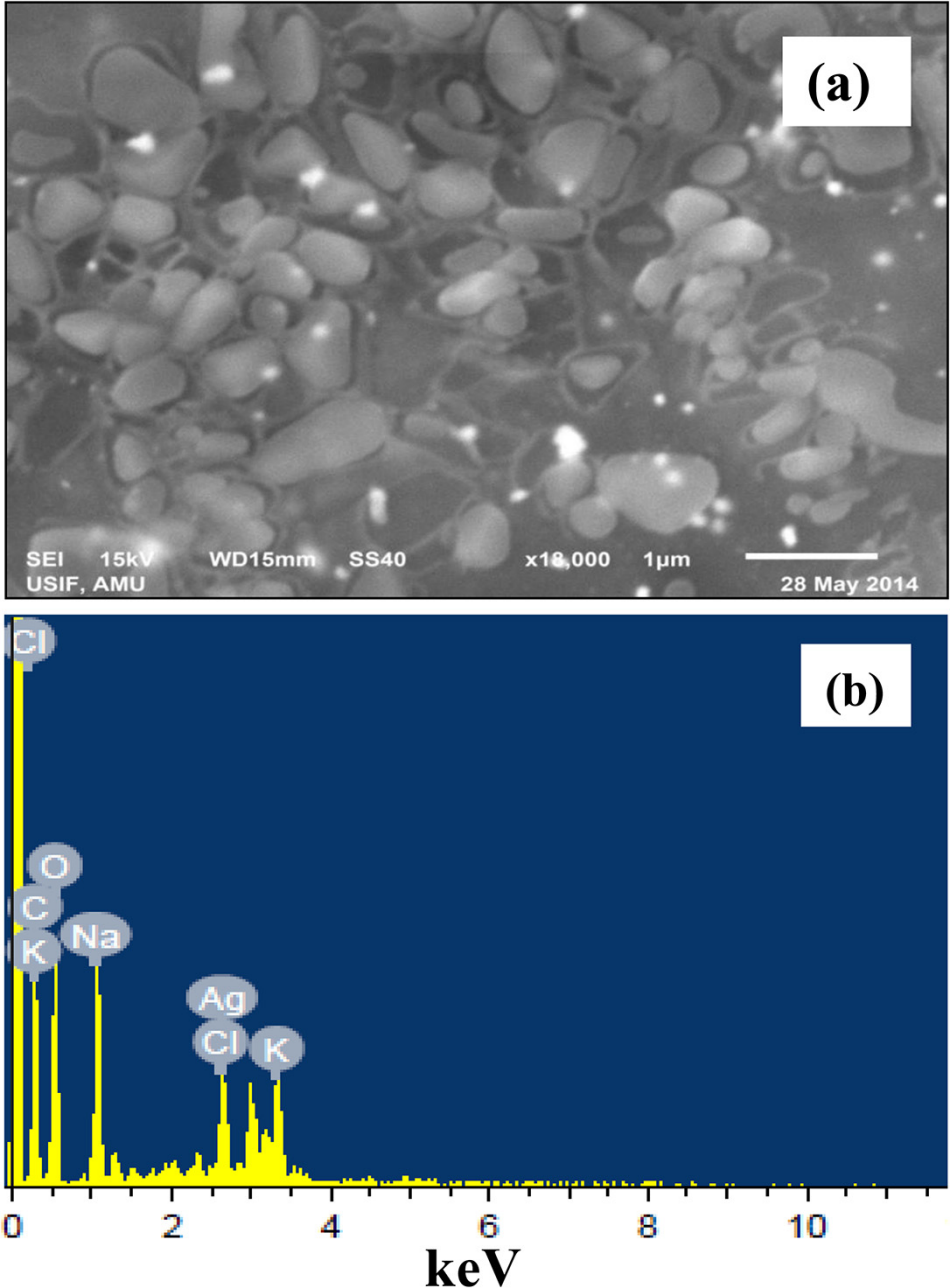
SEM and EDX analyses of ELE-AgNPs. Panel (a) shows the SEM images of ELE-AgNPs; Panel (b) represents the energy dispersive X-ray spectrum of ELE-AgNPs.

The TEM image (60,000 X, 200 Kv) showed variable and predominantly spherical ELE-AgNPs in size range of 1.9–4.3 nm and 5–25 nm with and without microwave assisted synthesis, respectively (Fig 5A and 5B). The scanning electron microscope images at 18,000X magnification exhibited that ELE-capped AgNPs were spherical and/or oval in shape with regular and smooth surface (Fig 6A). Fig 6B shows EDX spectrum of ELE-AgNPs, where a silver signal (12.89%) in the EDX spectrum exhibited the presence of elemental silver along with the signals of O, C, Al, Na, K, Cl, and K, as the components in the reaction medium. The feeble signal may possibly due to the biomolecules that are bound to the surface of AgNPs [14]. Thermogravimetric analysis (TGA) curve in S3 Fig. demonstrated thermal stability of ELE coated AgNPs in an inert atmosphere. The TGA plot of the capped AgNPs prepared using ELE showed a steady weight loss in the temperature range of 50–800°C. The results indicating weight loss of the ELE-AgNPs powder due to desorption of bioorganic compounds suggests a strong interaction between ELE and AgNPs.

**Fig 7:**
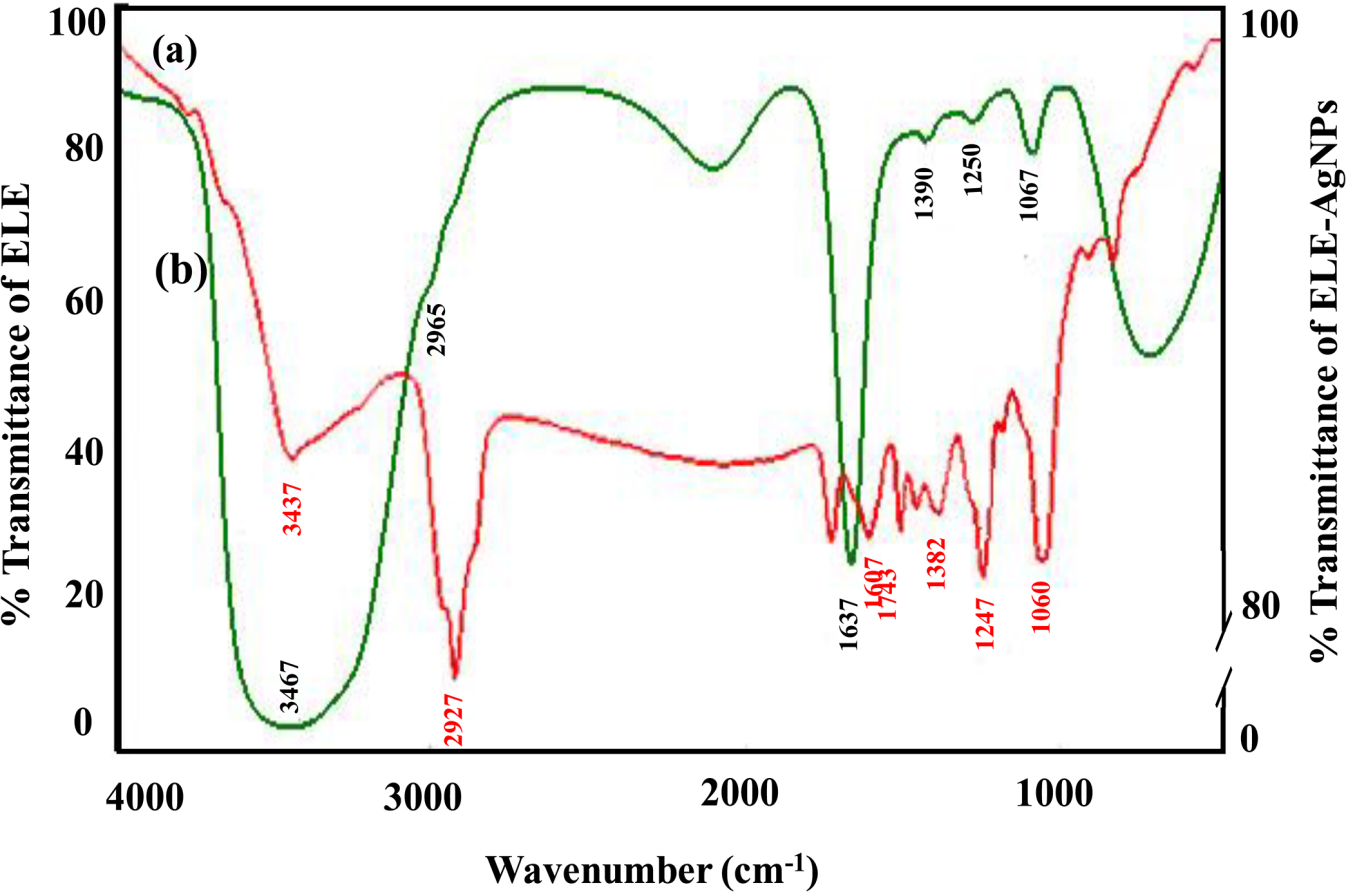
FTIR spectra of synthesized ELE-AgNPs. Panels (a) and (b) depict the spectra of ELE- AgNPs and ELE alone, respectively.

Although, the antibacterial ability of AgNPs has been adequately proven, their poor stability is a primary limitation to its further clinical application. Therefore, FTIR analysis have been performed to assess the role of various phytochemicals of *E*. *globulus* responsible for the synthesis and stabilization of AgNPs. Fig 7 shows the FTIR spectra of *E*. *globulus* stabilized AgNPs. A broad peak at about 3437 cm^-1^, is attributed to the stretching vibrations of hydroxyl (-OH) group. The weak absorption at 2927 cm^-1^ could be assigned to aliphatic C-H stretching vibrations, and the peak at 1743 cm^-1^ corresponds to carbonyl groups from dimerized saturated aliphatic acids. The absorption band at 1607 cm^-1^ can be attributed to C = C stretching vibrations of vibrations of aromatic ring. The peak at 1382 cm^-1^ may be due to O-H bending vibrations of polyols such as flavanoids present in leaf extract. The peak which appears at 1247 cm^-1^ may be due to C-O-H bending vibrations. The prominent peak at 1060 cm^-1^ corresponds to C-O stretching from alcohol, carboxylic acid, ester and ethers and C-N stretching vibration of the amine. All these vibration bands come from various alkaloids, flavanoids, and other phytochemicals, abundantly present in the leaf. A thin layer of biomaterials from the plant extract surrounding the ELE-AgNPs can be seen in the SEM image (Fig 6A). Gericke and Pinches [48] reported that the presence of carbonyl groups (>C = O) suggests that flavanones or terpenoids are also absorbed on the surface of metal NPs, possibly by interaction through carbonyl groups or π-electrons in the absence of other strong ligating agents in sufficient concentration. Several naturally occurring aromatic organic compound including terpenes and terpenoids such as 1,8-cineole (45.4%), limonene (17.8%), p-cymene (9.5%), c-terpinene (8.8%), a-pinene (4.2%) and a-terpineol (3.4%), while in the vapour, 1,8-cineole (34.6%), limonene (29.9%), p-cymene (10.5%), c-terpinene (7.4%), a-pinene (4.0%) and a-phellandrene (2.4%) are present in *E*. *globulus* plants [49]. Dubey et al. [16] have suggested that the flavanoid and terpenoid constituents present in *Eucalyptus hybrida* leaf extract are the surface active molecules stabilizing the AgNPs. The terpenoids may play a role in reduction of metal ions by oxidation of aldehydic groups in the molecules to carboxylic acids. Also, the presence of reducing sugars in the solution may also be responsible for the reduction of metal ions and formation of AgNPs [50]. Further, Paredes et al. [6] reported that certain amino acids could be used as reducing agents in the preparation of nanosilver. Besides, the carbonyl groups from the amino acid residues and proteins have stronger ability to bind metal indicating that the proteins could possibly provide capping of AgNPs to prevent agglomeration [46]. Indeed, stability is a critical factor when AgNPs are to be used for antibacterial treatment. In past decades, the approach most commonly used to address this problem involved modification of citrate on the surface of the nanosilver to obtain acceptable stability [8, 51]. Van der Zande et al. [10] reported improved stability of nanosilver after modification with polyvinylpyrrolidone in comparison with citrate coating. Thus, our results with ELE functionalized AgNPs demonstrated stability up to four weeks support the earlier studies and a recently study of Qu et al. [11] who have reported that AgNPs coated with *Agrimoniae herba* extract as the reducing agent and stabilizer, showed better stability and stronger pharmacological activity than bare AgNPs.

### Antibacterial and antibiofilm activity of ELE-AgNPs

**Fig 8:**
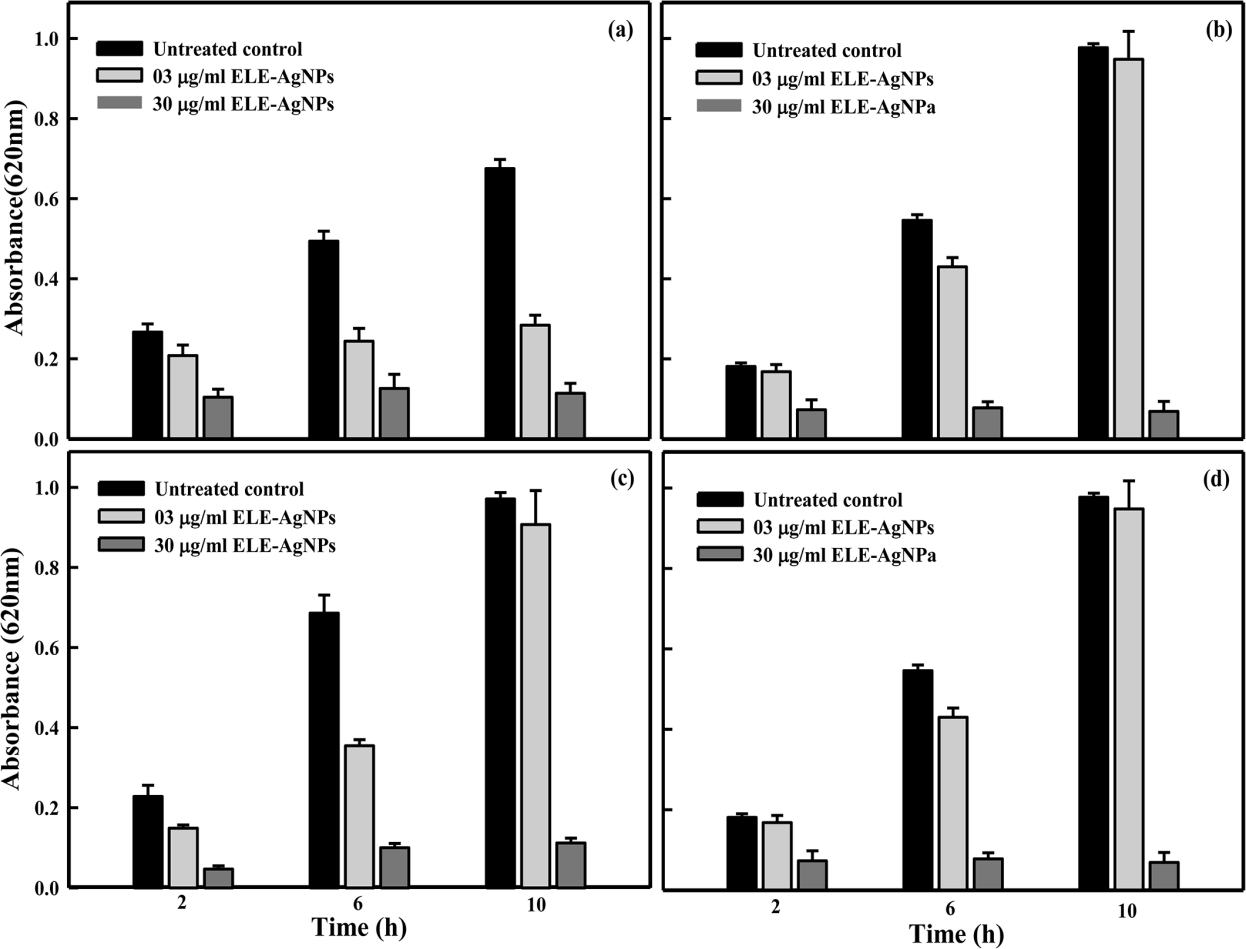
Antibacterial activity of ELE-AgNPs. Histogram showing change in absorbance of growth culture of clinical bacterial isolates at ELE-AgNPs concentrations of 3 and 30 μg/ml after 2, 6 and 10 h treatment at 37°C. Panel (a) Gram-negative (ESBL positive) *E*.*coli* (b) Gram-negative (ESBL positive) *P*. *aeruginosa;* (c) Gram-positive (methicillin-sensitive) *S*. *aureus* and (d) Gram-positive (methicillin-sensitive) *S*. *aureus*. The data represent the mean ± S.D of two independent experiments done in triplicate.

**Fig 9:**
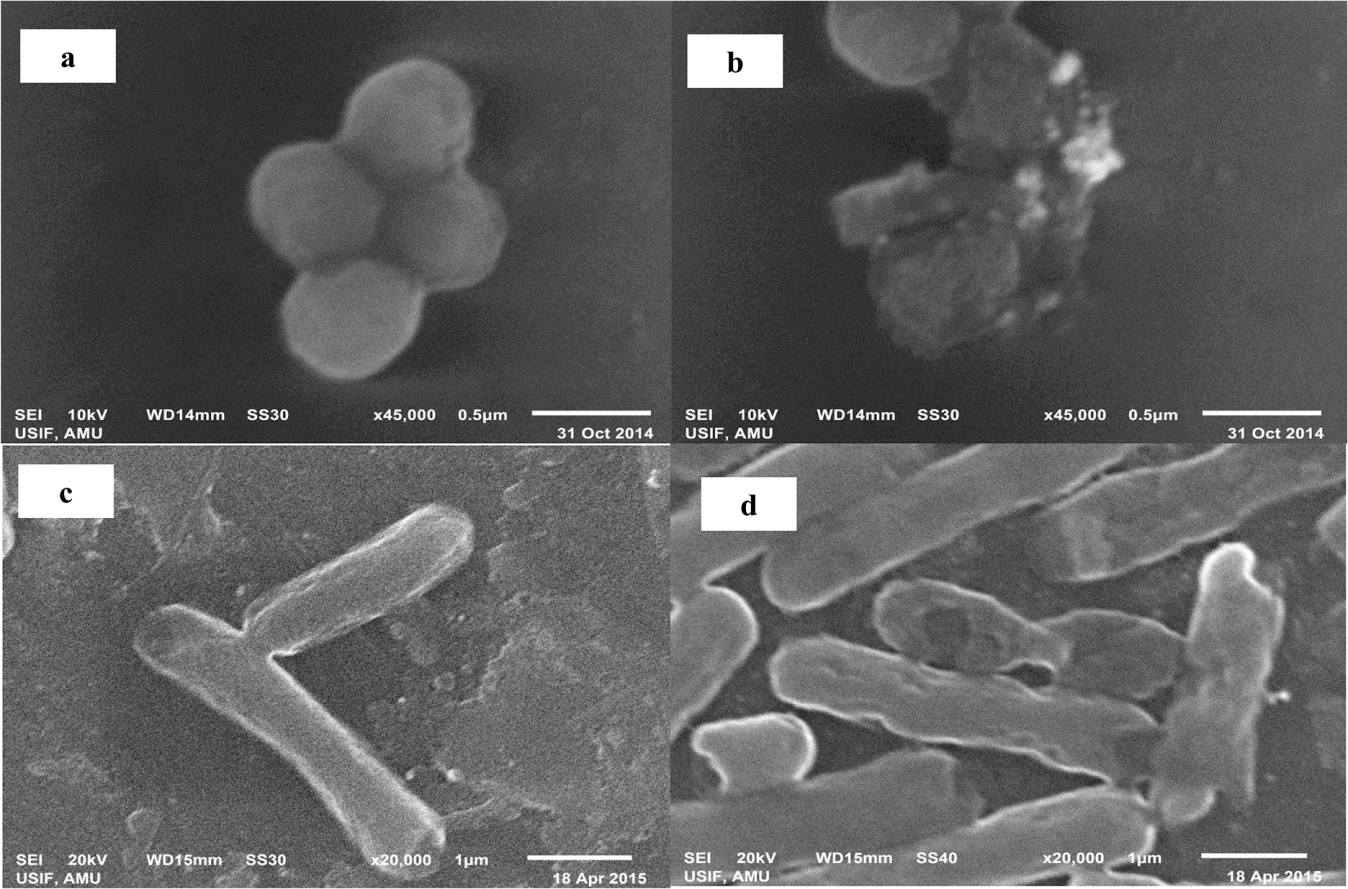
Interaction of ELE-AgNPs with bacterial cells. Representative SEM images showing the cellular damage and surface binding of ELE-AgNPs with (b) methicillin resistant *S*. *aureus* and (d) *E*.*coli* cells. The images in panels (a) and (c) show the untreated controls of *S*. *aureus* and *E*.*coli* cells, respectively.

The ELE-AgNPs were evaluated for their antimicrobial activity against ESBL producing *E*. *coli*, *P*. *aeruginosa* and methicillin sensitive and resistant *S*. *aureus*. The data in S4 Fig exhibit the dose dependent effect of ELE-AgNPs on the test strains in concentration range of 25–100μl, and suggested maximum cytotoxic effect with 100 μl of ELE-AgNPs with all test strains. However, 100 μl the ELE alone has shown very little cytotoxic effects. The results of well diffusion assay on the effect of ELE and ELE-AgNPs at a constant dose of 100 μl each on the ESBL producers, MRSA and MSSA strains are shown in S5 Fig Significant increase in the sizes of zones of inhibition (19–21 mm) with ELE-AgNPs, as compared to 8–10 mm, in case of extract alone, were observed with the test strains. The results suggested ELE-AgNPs induced growth inhibition in the order as *S*. *aureus* (MRSA) ≈ S. *aureus* (MSSA) ≈*P*. *aeruginosa* (ESBL) > *E*. *coli* (ESBL). These results were further validated by measuring inhibition of bacterial growth kinetics based on OD620 measurement in a concentration range of 3–30 μg/ml of ELE-AgNPs. The results presented in Fig 8A–8D demonstrate a differential growth inhibition pattern of the ESBL producing *E*. *coli*, *P*. *aeruginosa* and methicillin sensitive and resistant *S*. *aureus* with increasing concentrations of ELE-AgNPs. The Gram +ve cells (*S*. *aureus*) were found to be more sensitive and susceptible to ELE-AgNPs than Gram-ve cells (*E*. *coli*, *P*. *aeruginosa*). Within the category of Gram +ve cells, the MRSA were more sensitive (6% survival) to ELE-AgNPs induced cellular damage than MSSA (11% survival). While, amongst the ESBL producers, the *P*. *aeruginosa* cells were more sensitive (5% survival) than *E*. *coli* (10% survival) (Fig 8) at the greatest concentration of 30 μg/ml ELE-AgNPs after 10 h of incubation. Indeed, significant growth inhibition occurred after 2 h of treatment with ELE-AgNPs and not much increase in cytotoxicity ensued upon extended incubation up to 10 h. The MIC and MBC values ELE-AgNPs for the ESBL producing *P*. *aeruginosa* and *E*. *coli* were determined to be 27 and 36 μg/ml and 36 and 42μg/ml, respectively. Whereas, the MIC and MBC values ELE-AgNPs with MRSA and MSSA were estimated to be 27 and 30 μg/ml, and 30 and 33 μg/ml, under identical conditions (results not shown). The representative SEM images in Fig 9A–9D show the interaction of ELE-AgNPs with the of *S*. *aureus* and *E*. *coli* strains at a sub lethal concentration of 20 μg/ml, which resulted in cell surface binding of ELE-AgNPs and cellular damage, as compared to untreated control.

**Fig 10:**
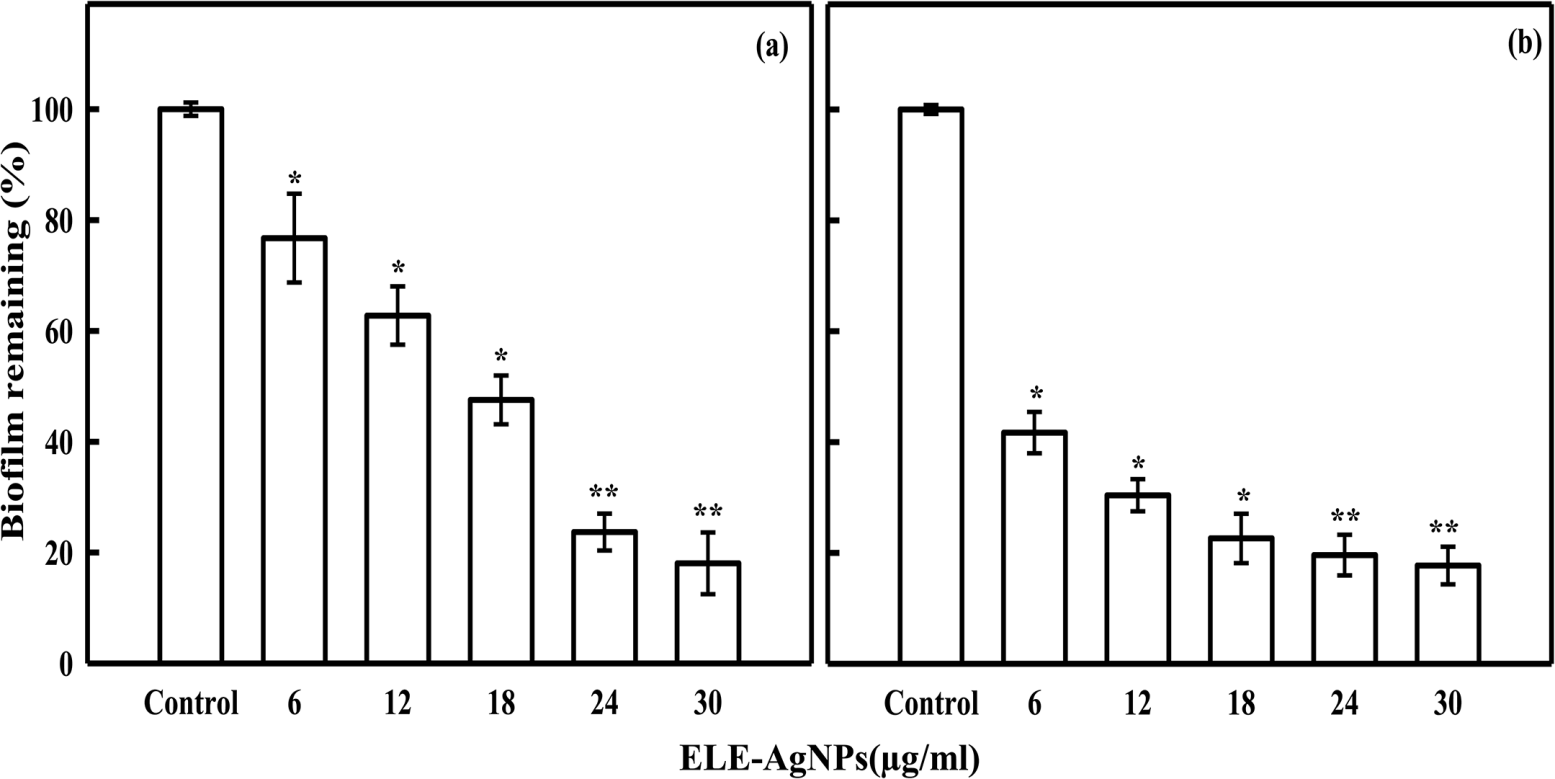
ELE-AgNPs concentration dependent inhibition of bacterial biofilm formation. Panels (a) and (b) show the inhibition of biofilm formation by *P*. *aeruginosa* (ESBL positive), and methicillin resistant *S*. *aureus* (MRSA), respectively. The data represent the mean ± S.D of two independent experiments done in triplicate. *p< 0.01; **p<0.001.

The results in Fig 10 show the ELE-AgNPs concentration dependent inhibition of biofilm formation. The data revealed 82 ± 3% and 81 ± 5% inhibition of biofilms formed by *S*. *aureus* and *P*. *aeruginosa*, respectively, at 30μg/ml after 24 h of treatment. Both the *S*. *aureus* and *P*. *aeruginosa* are known to be potent biofilm producers, which makes their clinical management difficult. They are regarded as causative agents of many infections in humans, ranging from superficial skin suppurations to life-threatening septicemias associated with visceral or bone infections [52]. Successful treatment becomes more challenging by the increasing prevalence of methicillin-resistance and antibiotic inefficacy, when such bacteria are involved in chronic infections. Thus, ELE-AgNPs induced quorum quenching could be an alternate strategy, apart from conventional antibiotic therapy, for combating biofilm based bacterial infections. Nevertheless, it could be argued that there are polyphenolic compounds, such as epigallocatechin gallate, ellagic acid and tannic acid present in plant leaf extracts, which may also acts like a strong siderophores, which chelate iron from the medium and thus causes the degradation of bacterial biofilm [53]. *Eucalyptus* leaves are reported to contain up to 11% of the major components of tannin (gallic acid and ellagic acid) with flavonoids (quercetin, rutin, etc.) as minor substances [54]. However, under our experimental conditions, the heat treatment of ELE at 90°C, most likely resulted in thermal degradation of tanins and other poly phenols. Rakić et al. [55], have reported the thermal degradation of tanins in oak acorn kernel *Quercus cerris* at 60°C. Therefore, no biofilm inhibition activity of ELE alone, as a control was observed, which suggests the role of ELE-AgNPs in significant inhibition of biofilm formed by *P*. *aeruginosa* and *S*. *aureus*. Kalishwaralal et al. [56], have also demonstrated a potential anti-biofilm activity of biologically synthesized AgNPs against the *P*. *aeruginosa* and *S*. *epidermidis*, which corroborate our findings.

Various NPs and their oxides such as iron, iron oxides, copper, copper oxides, titanium, titanium oxides, zinc, zinc oxides, silver and gold have already been used as potent antimicrobial agents. Silver being in nanoparticle or ionic form is most toxic for microorganisms when compared to other metals [20]. In fact, there is an ongoing debate regarding the role of released Ag^+^ ions from nanosilver and its toxicity against microorganisms [57]. Several studies have reported that dissolved Ag^+^ ions dictate nanosilver toxicity [58–59]. In contrast, many studies suggested that the effect of released Ag^+^ ions is not significant and the toxicity induced by nanosilver cannot be attributed solely to the released Ag^+^ ions but rather to nanosilver particles size, shape, surface coating and surface charge. These factors may affect toxicity indirectly through mechanism that influence the rate, extent, location and timing of Ag^+^ ions release [60–63]. Indeed, all these studies have employed commercially available nanosilver with a limitation on control over Ag size, morphology and degree of agglomeration. This makes difficult to draw universally accepted conclusions regarding the toxicity mechanism of nanosilver. Furthermore flocculation of nanosilver may occur in bacterial suspensions unless its surface is modified with surfactants or stabilizers, which may alter again the antibacterial activity of nanosilver. In our studies the ELE-AgNPs synthesized are possibly capped with the proteins/peptides, eucalyptol, terpinine, tannins, etc present in the plant extract, which may minimize the instant Ag^+^ ions leaching due to oxidative dissolution under oxic environment but may possibly go for slow and sustained release of Ag^+^ ions and therefore could serve as continuous source of Ag+ Ions, as also suggested by Dobias and Bernier-Latmani [64]. Sotiriou et al [65] have also demonstrated that when AgNPs are selectively conditioned by either washing or H_2_ reduction, the oxide layers are removed drastically minimizing Ag^+^ ions leaching and its antibacterial activity. Lately, Loza et al [66] have also demonstrated that if surface of NPs is passivated by cysteine, the dissolution is quantitatively inhibited.

Nanosized silver is reported to exert efficient toxicity to microbes but lower toxicity in mammalian cells [21]. Our data of MTT and Neutral red (NRU) cytotoxicity assay on cultured human breast carcinoma (MCF-7) cell line revealed 12% cytotoxicity at 30 μg/ml ELE-AgNPs, whereas the NRU data indicated only 10% toxicity in DMEM after 24 h incubation at 5% CO_2_ and 37°C (data not shown). In contrast, the results of ELE-AgNPs on bacteria indicated 47% killing of *P*. *aeruginosa* (ESBL positive), 38% growth inhibition of *E*.*coli* (ESBL positive), 43% inhibition of *S*. *aureus* (MSSA) and 37% inhibition of growth in *S*. *aureus* (MRSA) at concentration of 30 μg/ml ELE-AgNPs (Fig 8 and S6 Fig). Various hypotheses have been proposed to explain the mechanism of antimicrobial activity of AgNPs. It is widely believed that AgNPs are incorporated in the cell membrane, which causes leakage of intracellular materials and eventually causes cell death [20, 67–68]. Thus, it is concluded that the extract of *E*. *globulus* leaf are capable of producing stable AgNPs extracellularly. The organic constituents such as flavanoids and terpenes present in ELE are the surface active molecules stabilizing the AgNPs. Furthermore, the microwave assisted method is demonstrated to be a rapid and efficient approach involving environmentally benign and low cost reductant from a natural resource, besides providing a viable alternative to chemical protocols, for synthesizing AgNPs. The green synthesized ELE-AgNPs were found to be effective antibacterial and antibiofilm agents for *S*. *aureus* (MRSA and MSSA) and EMBL producing *P*. *aeruginosa* and *E*.*coli* clinical isolates. Further studies are warranted to optimize the conditions for large-scale production, and validation for determining the efficacy and dose response of ELE-AgNPs for clinical applications, as broad spectrum nanoantibiotics.

## Supporting Information

S1 Fig Effect of temperature on ELE-AgNPs synthesis. Panel A shows (a) 5 min microwave treatment at 800 W (2.45 GHz); (b) 10 min conventional heating at 60°C; (c) 60 min incubation at 37°C. Panel B shows the comparative synthesis of ELE-AgNPs as function of temperature by conventional heating and microwave treatment.
(TIF)

S2 Fig Stability assessment of ELE-AgNPs. UV-Vis spectra showing comparative analysis of ELE-AgNPs synthesized by microwave assisted green synthesis and chemically synthesized AgNPs by measuring the changes in surface plasmon resonance.
(TIF)

S3 Fig TGA thermogram of ELE-AgNO_3_. (TIF)

S4 Fig Quantitative analysis of antibacterial activity of ELE and ELE-AgNPs. Comparative antibacterial effects induced by 100 μl ELE as control and with increasing amounts (25–100 μl) of ELE-AgNPs against the MSSA, MRSA and ESBL-positive and-negative clinical bacterial isolates.
(TIF)

S5 Fig Assessment of antibacterial activity of ELE and ELE-AgNPs by well diffusion assay. (TIF)

S6 Fig Antibacterial activity of ELE-AgNPs. The effect of ELE-AgNPs concentration indicates differential growth inhibition patterns with different clinical bacterial isolates as a function of time. Panel (a) Gram-negative (ESBL positive) *E*. *coli* (b) Gram-negative (ESBL positive) *P*. *aeruginosa*; (c) Gram-positive (methicillin-sensitive) *S*. *aureus* and (d) Gram-positive (methicillin-sensitive) *S*. *aureus*. The data represent the mean ± S.D of two independent experiments done in triplicate.
(TIF)
