# Supplementary figures and images for: Microwave Accelerated Green Synthesis of Stable Silver Nanoparticles with Eucalyptus globulus Leaf Extract and Their Antibacterial and Antibiofilm Activity on Clinical Isolates
Source: PLoS One. 2015 Jul 1;10(7):e0131178. doi: 10.1371/journal.pone.0131178 (PMC4489395; doi:10.1371/journal.pone.0131178)

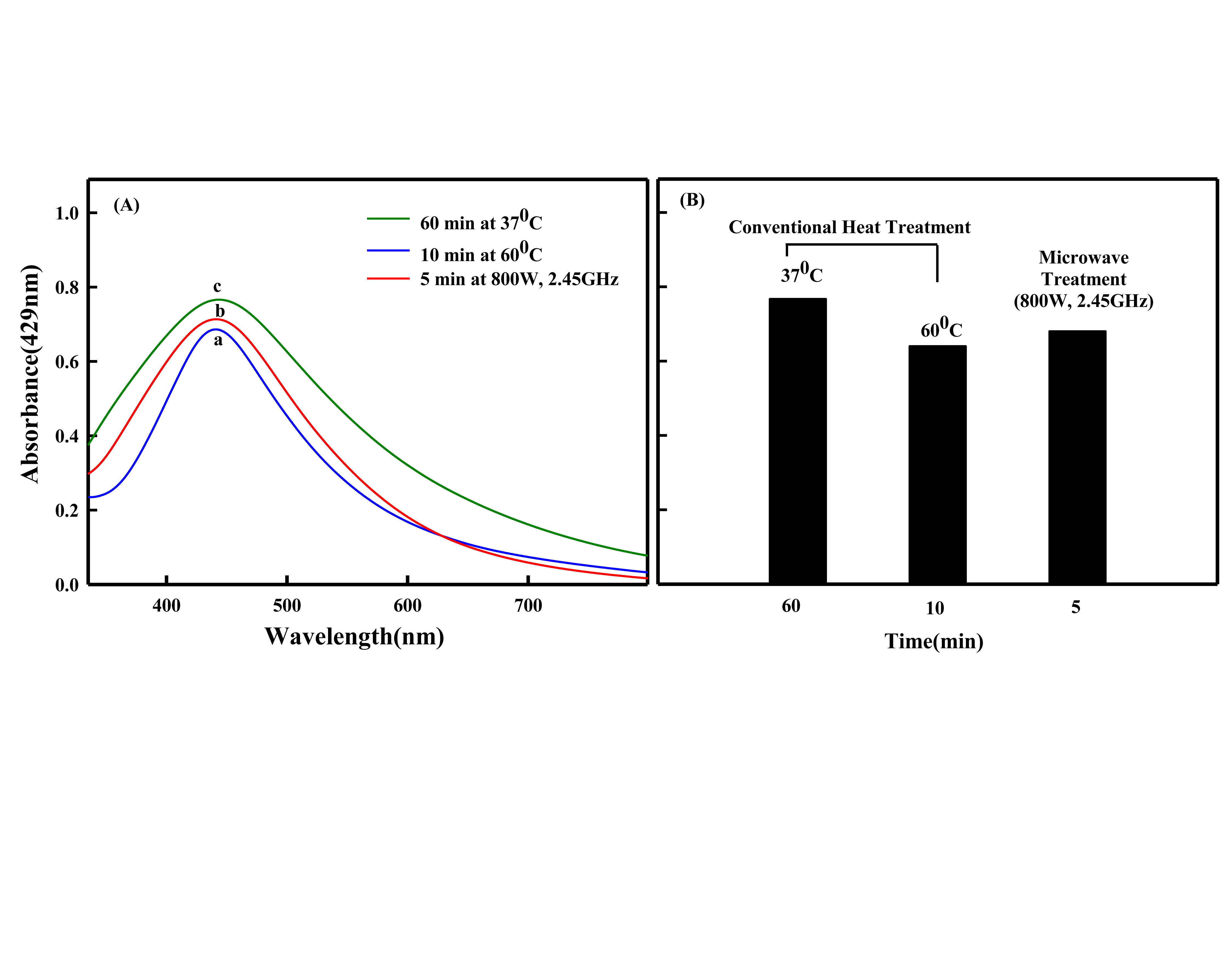

Supplement: S1 Fig — Panel A shows (a) 5 min microwave treatment at 800 W (2.45 GHz); (b) 10 min conventional heating at 60°C; (c) 60 min incubation at 37°C. Panel B shows the comparative synthesis of ELE-AgNPs as function of temperature by conventional heating and microwave treatment. (TIF) [file pone.0131178.s001.tif]

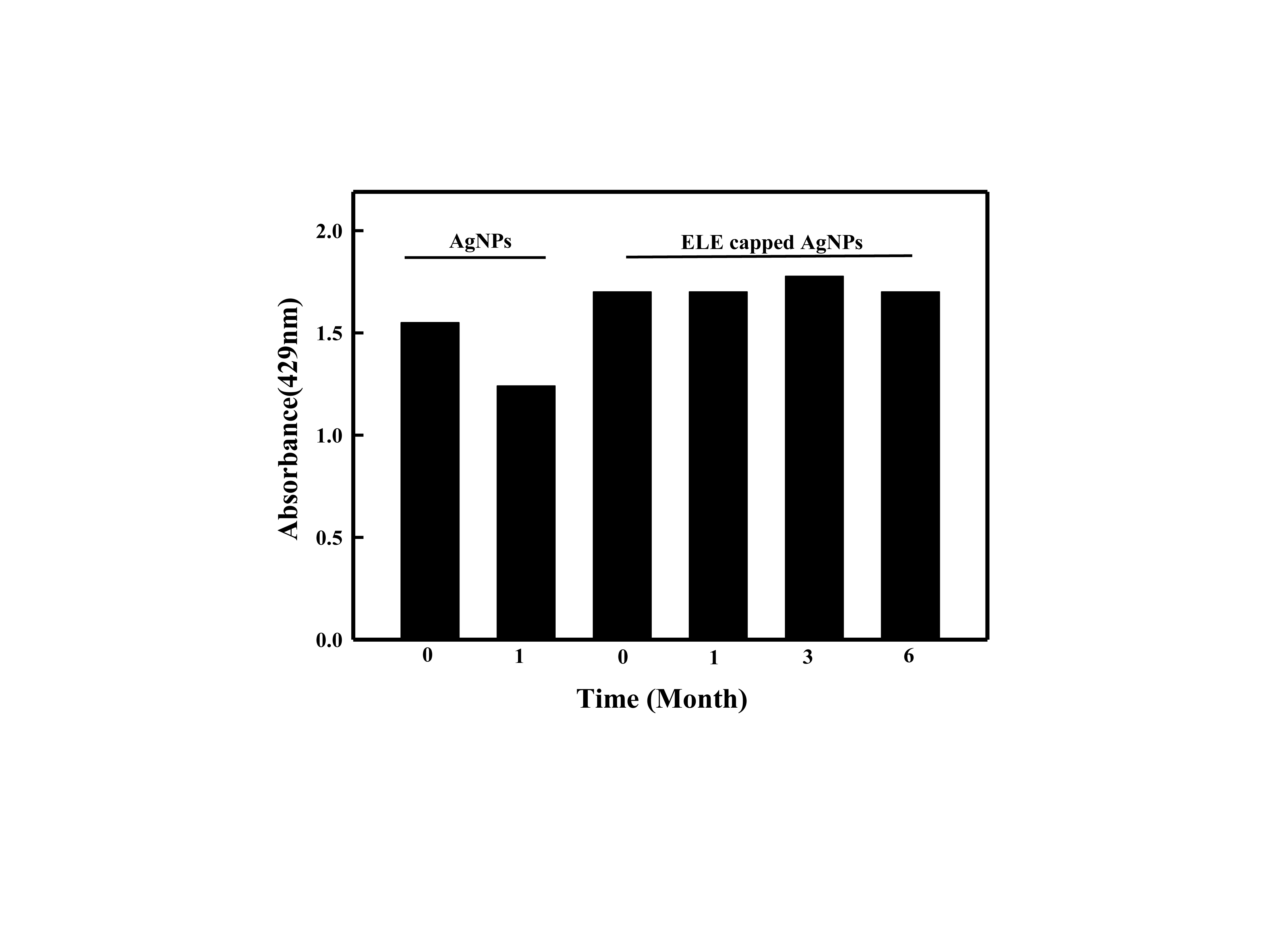

Supplement: S2 Fig — UV-Vis spectra showing comparative analysis of ELE-AgNPs synthesized by microwave assisted green synthesis and chemically synthesized AgNPs by measuring the changes in surface plasmon resonance. (TIF) [file pone.0131178.s002.tif]

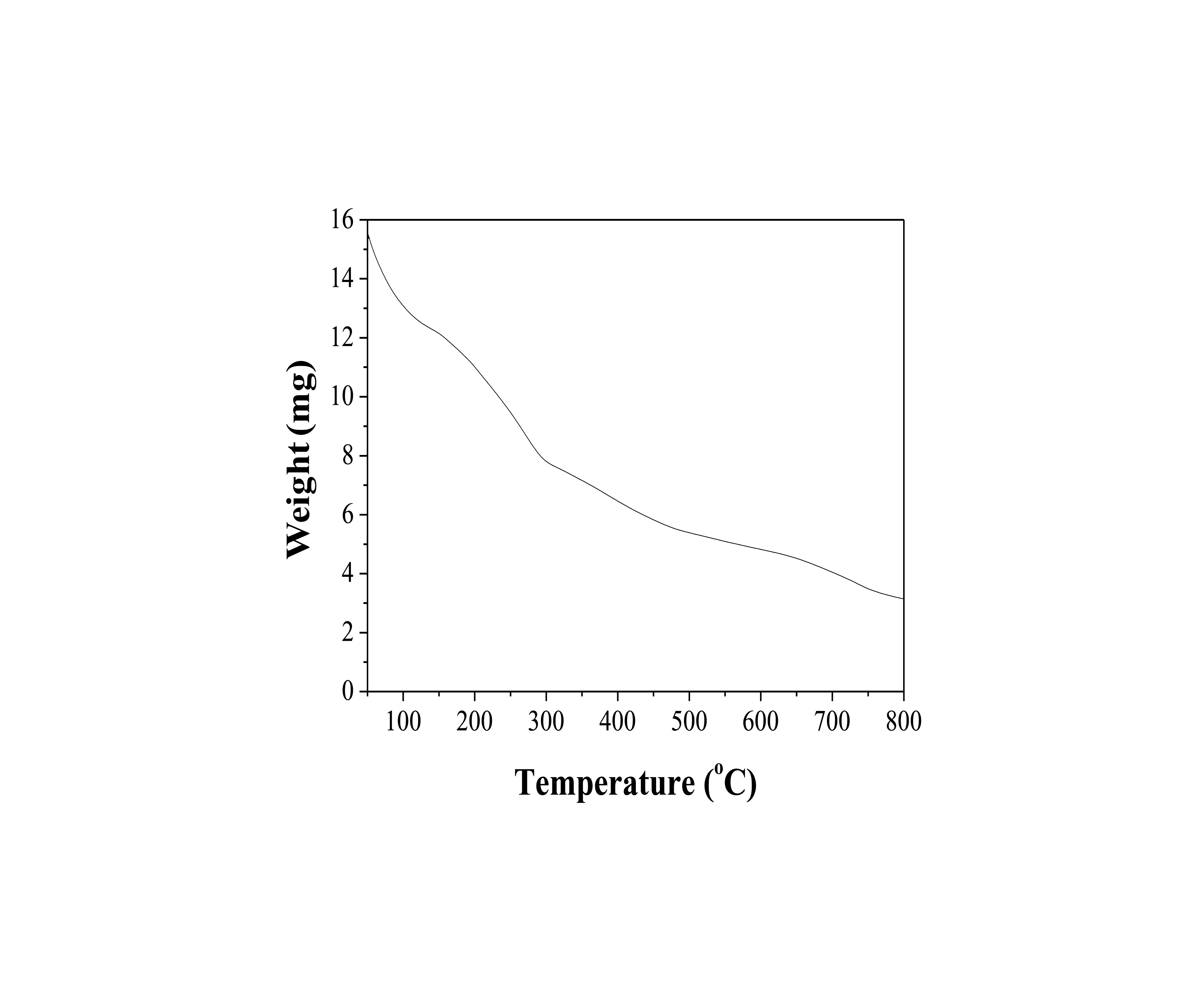

Supplement: S3 Fig — (TIF) [file pone.0131178.s003.tif]

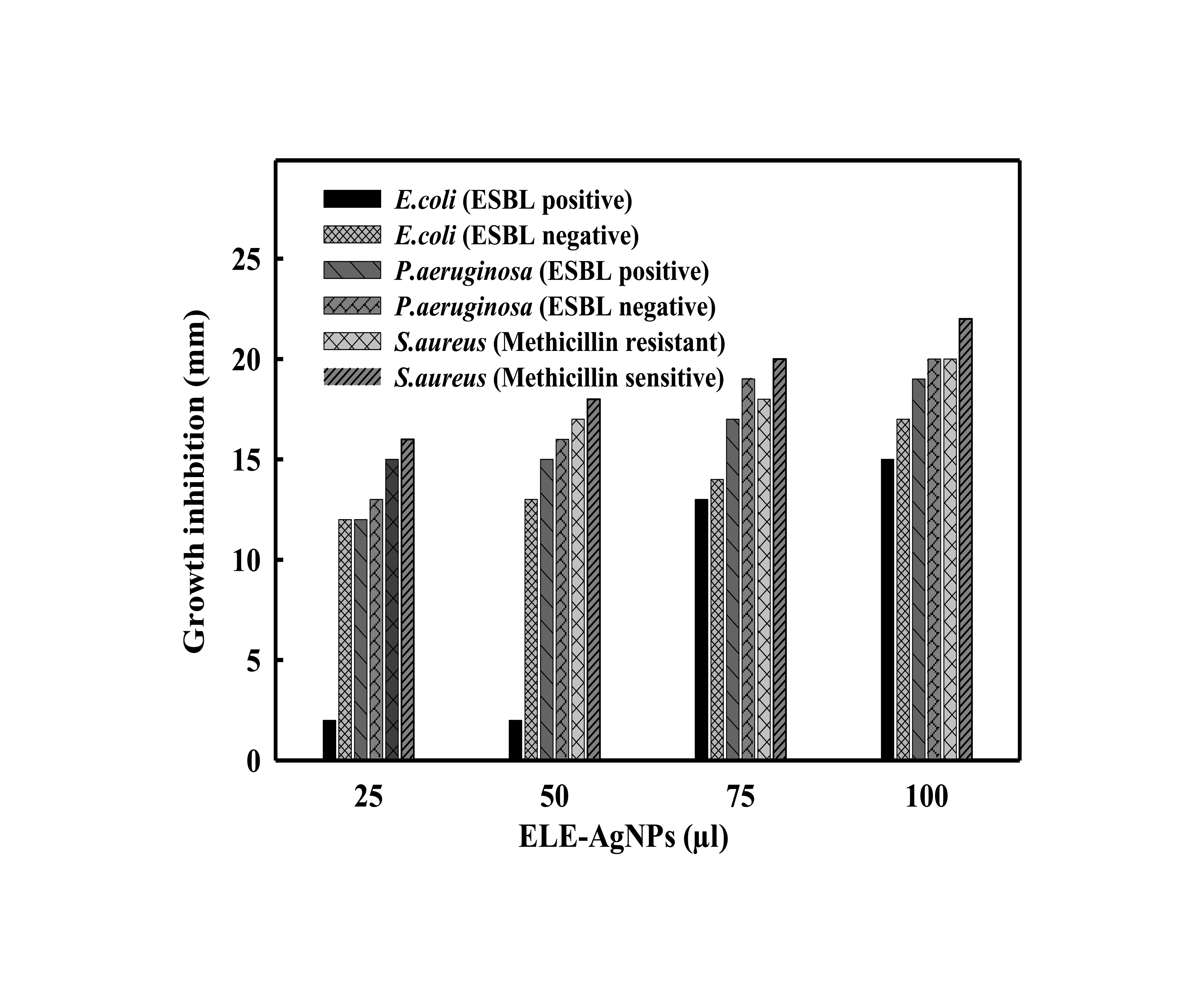

Supplement: S4 Fig — Comparative antibacterial effects induced by 100 μl ELE as control and with increasing amounts (25–100 μl) of ELE-AgNPs against the MSSA, MRSA and ESBL-positive and-negative clinical bacterial isolates. (TIF) [file pone.0131178.s004.tif]

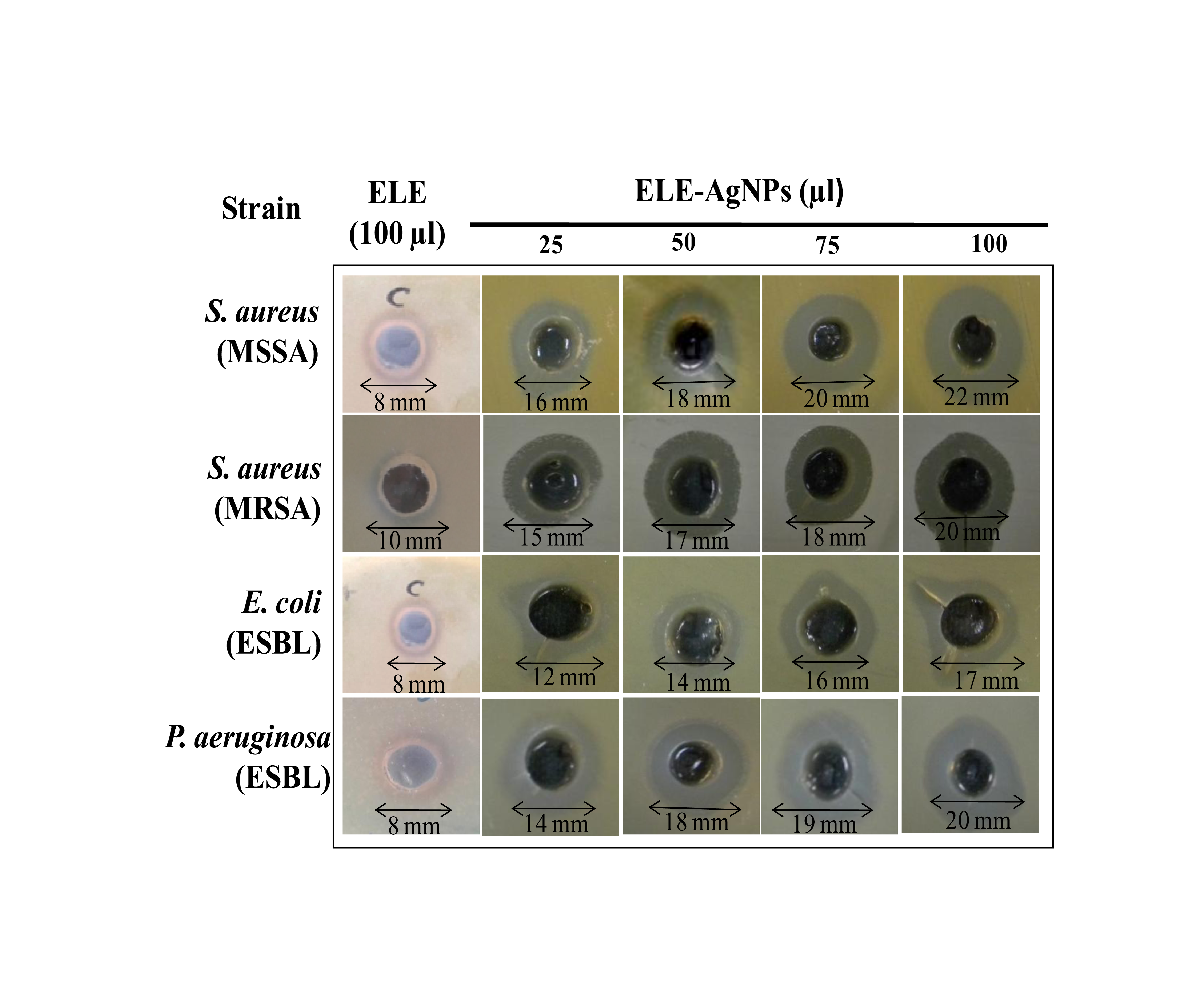

Supplement: S5 Fig — (TIF) [file pone.0131178.s005.tif]

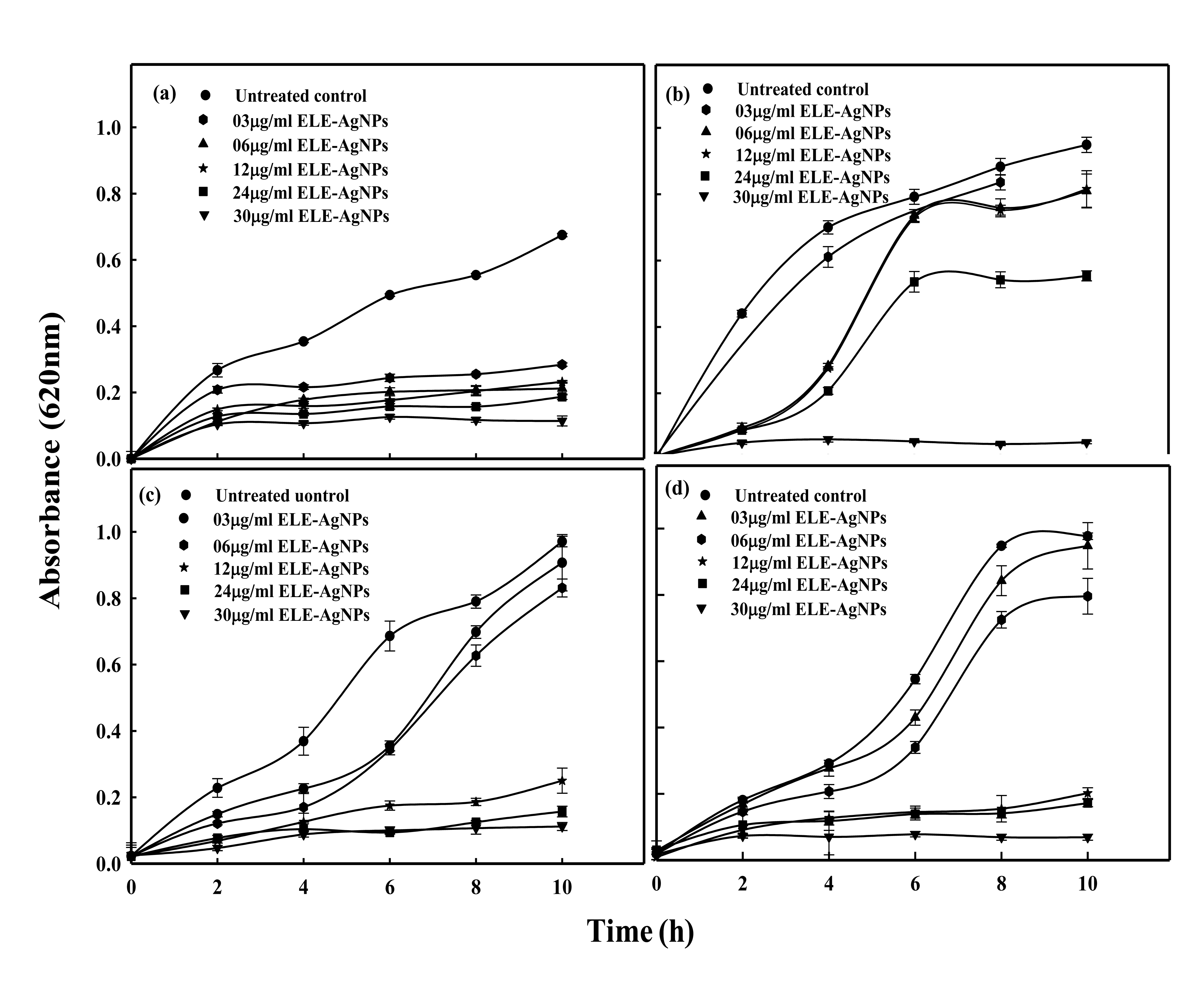

Supplement: S6 Fig — The effect of ELE-AgNPs concentration indicates differential growth inhibition patterns with different clinical bacterial isolates as a function of time. Panel (a) Gram-negative (ESBL positive) E. coli (b) Gram-negative (ESBL positive) P. aeruginosa; (c) Gram-positive (methicillin-sensitive) S. aureus and (d) Gram-positive (methicillin-sensitive) S. aureus. The data represent the mean ± S.D of two independent experiments done in triplicate. (TIF) [file pone.0131178.s006.tif]
